# Altered resting‐state hippocampal and caudate functional networks in patients with obstructive sleep apnea

**DOI:** 10.1002/brb3.994

**Published:** 2018-05-10

**Authors:** Xiaopeng Song, Bhaswati Roy, Daniel W. Kang, Ravi S. Aysola, Paul M. Macey, Mary A. Woo, Frisca L. Yan‐Go, Ronald M. Harper, Rajesh Kumar

**Affiliations:** ^1^ Department of Anesthesiology University of California at Los Angeles Los Angeles CA USA; ^2^ UCLA School of Nursing University of California at Los Angeles Los Angeles CA USA; ^3^ Department of Medicine University of California at Los Angeles Los Angeles CA USA; ^4^ Department of Neurology University of California at Los Angeles Los Angeles CA USA; ^5^ Department of Neurobiology University of California at Los Angeles Los Angeles CA USA; ^6^ Brain Research Institute University of California at Los Angeles Los Angeles CA USA; ^7^ Department of Radiological Sciences University of California at Los Angeles Los Angeles CA USA; ^8^ Department of Bioengineering University of California at Los Angeles Los Angeles CA USA

**Keywords:** anxiety, caudate nucleus, depression, functional magnetic resonance imaging, hippocampus, resting‐state functional connectivity, sleep disordered breathing

## Abstract

**Introduction:**

Brain structural injury and metabolic deficits in the hippocampus and caudate nuclei may contribute to cognitive and emotional deficits found in obstructive sleep apnea (OSA) patients. If such contributions exist, resting‐state interactions of these subcortical sites with cortical areas mediating affective symptoms and cognition should be disturbed. Our aim was to examine resting‐state functional connectivity (FC) of the hippocampus and caudate to other brain areas in OSA relative to control subjects, and to relate these changes to mood and neuropsychological scores.

**Methods:**

We acquired resting‐state functional magnetic resonance imaging (fMRI) data from 70 OSA and 89 healthy controls using a 3.0‐Tesla magnetic resonance imaging scanner, and assessed psychological and behavioral functions, as well as sleep issues. After standard fMRI data preprocessing, FC maps were generated for bilateral hippocampi and caudate nuclei, and compared between groups (ANCOVA; covariates, age and gender).

**Results:**

Obstructive sleep apnea subjects showed significantly higher levels of anxiety and depressive symptoms over healthy controls. In OSA subjects, the hippocampus showed disrupted FC with the thalamus, para‐hippocampal gyrus, medial and superior temporal gyrus, insula, and posterior cingulate cortex. Left and right caudate nuclei showed impaired FC with the bilateral inferior frontal gyrus and right angular gyrus. In addition, altered limbic‐striatal‐cortical FC in OSA showed relationships with behavioral and neuropsychological variables.

**Conclusions:**

The compromised hippocampal‐cortical FC in OSA may underlie depression and anxious mood levels in OSA, while impaired caudate‐cortical FC may indicate deficits in reward processing and cognition. These findings provide insights into the neural mechanisms underlying the comorbidity of mood and cognitive deficits in OSA.

## 1 INTRODUCTION

Obstructive sleep apnea (OSA) is common condition, affecting as much as 10% of the population (Lee, Nagubadi, Kryger, & Mokhlesi, 2008), and is characterized by recurrent episodes of complete or partial obstruction of the upper airway, with continued diaphragmatic efforts to breathe during sleep. The pattern leads to episodic O_2_ desaturations accompanied by significant blood pressure changes (Lee et al., 2008). In addition to physiological symptoms, OSA patients often show several neuropsychological comorbidities, including cognitive deficits, depression, and anxiety symptoms (Kumar et al., 2009; Sforza, de Saint Hilaire, Pelissolo, Rochat, & Ibanez, 2002). Several studies have shown that OSA contributes to cognitive and mood decline, affecting 17%–41% of OSA adult patients (Asghari, Mohammadi, Kamrava, Tavakoli, & Farhadi, 2012; DeZee, Hatzigeorgiou, Kristo, & Jackson, 2005; Harris, Glozier, Ratnavadivel, & Grunstein, 2009; Sharafkhaneh, Giray, Richardson, Young, & Hirshkowitz, 2005). Increased prevalence of OSA in several psychiatric disorders has been reported, with major depressive disorder, anxiety, and post‐traumatic stress disorder having a higher incidence (Gupta & Simpson, 2015; Harris et al., 2009). If left untreated, OSA can result in altered cognition with reduced attention, memory, and executive functioning, and reduced quality of life (Rosenzweig et al., 2015). Although the pathological basis for underlying neuropsychological comorbidities in OSA are poorly understood, earlier studies have proposed several mechanisms, including brain tissue injury induced by intermittent hypoxia or ischemia, and emotion‐related network dysfunction caused by sleep disturbances and fragmented sleep (Goldstein & Walker, 2014; Rosenzweig et al., 2015; Sforza & Roche, 2012).

Cognitive and emotional deficits in OSA subjects may arise from brain injury and oxidative and neuro‐inflammatory effects on regulatory brain sites for those functions (Rosenzweig, Williams, & Morrell, 2014; Rosenzweig et al., 2015; Sforza & Roche, 2012). A substantial number of MRI studies have shown that OSA is accompanied by a range of tissue changes, including altered white matter integrity, free water content, brain metabolites, and regional gray matter volume changes. The affected structures include the hippocampus, basal ganglia, thalamus, cingulate, insula, medulla, cerebellar, and frontal regions (Kumar et al., 2012, 2014; Macey et al., 2008). Several of these sites play significant roles in autonomic regulation, and in addition, cognitive and affective control. Intermittent hypoxia and recurrent hypoxemia accompany OSA, and can affect neuronal cells, axons, and glia directly (Almendros, Wang, & Gozal, 2014; Douglas et al., 2007; Gozal, Row, Schurr, & Gozal, 2001). In addition, hypoxia triggers endothelial cell dysfunction that can compromise the blood–brain barrier, as shown in OSA (Palomares et al., 2015); a long‐term breakdown in the blood–brain barrier can promote tissue injury (Almendros et al., 2014).

Sleep deficits and fragmented rapid eye movement (REM) sleep can alter molecular signaling pathways that regulate synaptic strength, plasticity‐related gene expression, and protein translation in mood and cognition regulatory circuitries (Abel, Havekes, Saletin, & Walker, 2013). Frequent brief awakenings, and the ensuing fragmented sleep in OSA affect cognitive and emotional functions in a similar manner to total sleep deprivation (Rosenzweig et al., 2015). Sleep disturbances can impair neuronal excitability and decrease myelination, and may lead to cellular oxidative stress, and mis‐folding of cellular proteins in mood and cognitive regulatory networks (Abel et al., 2013; Picchioni, Reith, Nadel, & Smith, 2014). A close association exists between oxidative stress, sleepiness, and the presence of affective symptoms in OSA patients (Franco et al., 2012).

At the regional level, brain structural injury and metabolic deficits in two subcortical structures, hippocampus and caudate nuclei, have been linked to negative emotions, including depression and anxiety in OSA (Cross et al., 2008; Dedovic et al., 2016; Kumar et al., 2009; Langenecker, Jacobs, & Passarotti, 2014). Hippocampal and caudate nuclei are structurally impaired in both adult and child OSA subjects (Halbower et al., 2006), and show enhanced damage in OSA subjects with mood symptoms (Cross et al., 2008; Kumar et al., 2009). Routine magnetic resonance imaging (MRI) has shown white matter infarcts and gray matter tissue loss in the hippocampus and caudate nuclei, as well as in cortical areas (Macey et al., 2008; Morrell et al., 2003). Reduced brain metabolites, including *N*‐acetyl aspartate and choline also appear in these regions in adult and pediatric OSA (Bartlett et al., 2004; Halbower et al., 2006). These findings reflect regional neuronal cell loss and significant morphologic and metabolic changes in untreated OSA subjects induced by hypoxia and neuro‐inflammatory responses. However, how these local sites interact with cortical areas during resting‐states to mediate affective symptoms and cognitive deficits at the network level in OSA are unclear.

Resting‐state functional MRI techniques have been widely applied in various clinical studies of psychiatric symptoms and other neurological issues (Hu, Song, Li, et al., 2015; Hu, Song, Yuan, et al., 2015; Song et al., 2015), and thus, may be useful in assessing network‐level changes in OSA subjects. Functional connectivity (FC), a measurement that quantifies statistical dependency between the functional time series of anatomically distinct brain sites, is a robust way to assess functional network integrity.

Our aim was to investigate how the hippocampus and caudate nuclei interact during resting‐states with other brain regions in newly diagnosed, treatment‐naïve OSA patients, relative to age‐ and sex‐comparable control subjects using resting‐state FC procedures. We hypothesized that hippocampal and caudate nuclei interactions to other mood and cognitive regulatory areas would be compromised in OSA subjects, and that these altered functional connections will show associations with sleep and neuropsychological scores.

## 2 MATERIALS AND METHODS

### 2.1 Subjects

We recruited 70 newly diagnosed, treatment‐naïve OSA subjects from the Sleep Disorders Laboratory at the University of California at Los Angeles (UCLA) Medical Center and 89 age‐ and gender‐comparable healthy controls. Demographic, sleep, mood, cognitive, and physiologic data of OSA and control subjects are summarized in Table 1. OSA subjects were diagnosed based on overnight polysomnography (PSG). The PSG data were collected with Nihon Kohden system (https://www.nihonkohden.com, Tokyo, Japan), and scorings were manually performed by the sleep physicians. Detailed PSG data and sleep architecture of OSA subjects are provided in Table S1. We used standardized scoring criteria recommended by the American Academy of Sleep Medicine (AASM, 1999) to define OSA severity based on apnea‐hypopnea index (AHI), which includes both apneas and hypopneas, and is derived by dividing the number of apnea and hypopnea events by the total sleep time (“Sleep‐related breathing disorders in adults: recommendations for syndrome definition and measurement techniques in clinical research. The Report of an American Academy of Sleep Medicine Task Force,” 1999). Based on these criteria, OSA subjects were categorized as follows: mild OSA, AHI of 5 or more, but fewer than 15 events/hr; moderate OSA, AHI of 15 or more, but fewer than 30 events/hr; and severe OSA, AHI of 30 or more events/hr. Of 70 OSA subjects, seven had mild OSA, 28 moderate OSA, and 35 severe OSA. All subjects were medication‐free for any cardiovascular‐altering or mood‐regulation drugs. OSA and control subjects had no diagnosed history of neurological illness or psychiatric disorders. Control subjects were recruited through advertisements from the UCLA campus and Los Angeles area. We interviewed control subjects, as well as their sleep partners when available, to determine the potential for sleep disordered breathing, and subjects suspected of having such disturbed patterns, based on symptoms of snoring and gasping or with abnormal Pittsburgh Sleep Quality Index (PSQI) and Epworth Sleepiness Scale (ESS) scores, were excluded from the imaging study and underwent for an overnight PSG study. Any subject with a metallic implant or condition contraindicated for the MRI scanner environment was excluded. All participants gave written informed consent before MRI scanning or other data collection, and the study protocol was approved by the Institutional Review Board at the UCLA.

**Table 1:** Demographic, sleep, mood, and cognitive variables of OSA and control subjects

Variables	OSA (*n* = 70)	Controls (*n* = 89)	*p* values
Age range (years)	31–70	29–65	–
Mean age (years)	48.3 ± 9.2	46.4 ± 9.2	.24
Gender (Male:Female)	52:18	61:28	.63
BMI (kg/m^2^)	31.1 ± 6.2	24.8 ± 3.5	<.001
AHI (events/hr)	36.0 ± 23.3	–	–
BAI	9.3 ± 10.9	3.8 ± 4.9	<.001
BDI‐II	8.3 ± 8.0	3.8 ± 4.9	<.001
PSQI	8.7 ± 4.1	3.8 ± 2.5	<.001
ESS	9.8 ± 4.9	5.3 ± 3.6	<.001
Global MoCA	26.6 ± 3.6 (*n* = 45)	27.0 ± 3.2 (*n* = 46)	.235
MoCA: Visuospatial	4.0 ± 1.1 (*n* = 45)	4.6 ± 0.8 (*n* = 46)	<.01
MoCA: Naming	2.9 ± 0.2 (*n* = 45)	2.9 ± 0.2 (*n* = 46)	.62
MoCA: Attention	5.4 ± 1.0 (*n* = 45)	5.4 ± 0.3 (*n* = 46)	.75
MoCA: Language	2.7 ± 0.8 (*n* = 45)	2.5 ± 0.8 (*n* = 46)	.25
MoCA: Abstraction	2.0 ± 0.5 (*n* = 45)	1.9 ± 0.3 (*n* = 46)	.14
MoCA: Delayed recall	3.6 ± 0.5 (*n* = 45)	3.7 ± 1.3 (*n* = 46)	.70
MoCA: Orientation	6.0 ± 0.2 (*n* = 45)	6.0 ± 0.3 (*n* = 46)	.79

AHI, apnea‐hypopnea index; BAI, beck anxiety inventory; BDI‐II, beck depression inventory II; BMI, body mass index; ESS, Epworth Sleepiness Scale; MoCA, montreal cognitive assessment; OSA, obstructive sleep apnea; PSQI, Pittsburgh Sleep Quality Index.

### 2.2 Assessment of sleep, mood, and cognition

We assessed sleep quality and daytime sleepiness in all OSA and control subjects using the ESS and PSQI self‐administered questionnaires, respectively. Depressive symptoms were evaluated by the Beck Depression Inventory II (BDI‐II), and anxiety symptoms by the Beck Anxiety Inventory (BAI) in both groups. The BDI‐II and BAI self‐administered questionnaires include 21 questions (each question score ranges from 0 to 3), with total scores ranging from 0 to 63, based on mood or anxiety symptoms. In a subset of 45 OSA and 46 healthy control subjects, we used the Montreal Cognitive Assessment (MoCA) test for cognitive assessment. Cognitive domains, including attention, visuospatial/executive functions, memory, language, visuospatial skills, conceptual thinking, calculations, and orientation, were examined using the MoCA test.

### 2.3 Magnetic resonance imaging

All participants underwent brain structural and functional imaging in a 3.0‐Tesla MRI scanner (Siemens, Magnetom Tim‐Trio, Erlangen, Germany). We used foam pads on either side of the head to minimize head motion during scanning. Resting‐state (Rs) fMRI data were collected with an echo planar imaging (EPI)‐based pulse sequence in the axial plane (repetition time [TR] = 2,000 ms; echo time [TE] = 30 ms; flip angle [FA] = 90°; field‐of‐view [FOV] = 230 × 230 mm^2^; matrix size = 64 × 64; slice thickness = 4.2 mm; volumes = 59). During the rs‐fMRI scanning, all subjects were instructed to rest with their eyes open, without focusing on any specific thoughts. High‐resolution T1‐weighted images were acquired using a magnetization prepared rapid acquisition gradient‐echo (MPRAGE) pulse sequence (TR = 2,200 ms; TE = 2.2, 2.34 ms; FA = 9°; FOV = 230 × 230 mm^2^; matrix size = 256 × 256, 320 × 320; voxel size = 0.9 × 0.9 × 1.0 mm^3^, 0.72 × 0.72 × 0.9 mm^3^). Proton density (PD) and T2‐weighted images were also obtained, using a dual‐echo turbo spin‐echo pulse sequence (TR = 10,000 ms; TE1, 2 = 17, 134 ms; FA = 130°; matrix size = 256 × 256; FOV = 230 × 230 mm^2^; slice thickness = 4.0 mm) in the axial plane.

### 2.4 Data preprocessing and FC analysis

We first examined anatomical scans for any serious brain pathology using high‐resolution T1‐weighted, PD‐, and T2‐weighted images of all subjects. We also assessed rs‐fMRI data for imaging or head motion‐related artifacts before data preprocessing. None of the subjects included here showed any serious brain injury, or head motion‐related or other imaging artifacts.

Prior to FC analysis, fMRI data were preprocessed using DPARSFA and SPM12 software: the initial three brain volumes were discarded to avoid signal saturation issues; the remaining 56 volumes were realigned to eliminate potential head‐motion, and co‐registered to T1‐weighted images. The time series of each voxel was then band‐pass filtered (0.01–0.08 Hz), and effects of six rigid‐body motion parameters, their first and second derivatives, and global white matter, cerebrospinal fluid, and global brain signal changes were regressed. Images were then spatially normalized to a canonical space template using nonlinear transformation procedures, and spatially smoothed with a 4‐mm full‐width at half‐maximum Gaussian kernel. Averaged cortical maps, derived from T1‐weighted images of OSA and controls, and averaged whole‐brain T1‐weighted images, calculated from normalized T1‐weighted images of all OSA and controls, were used for anatomical references.

We applied canonical signal processing procedures to calculate the resting‐state FC for each rs‐fMRI dataset. The left and right hippocampus, as well as caudate, were selected as seed regions. Functional connectivities between regional mean time series for seed regions and whole‐brain voxels were calculated with a canonical correlation approach. A positive correlation between the mean time course of the seed region and a specific brain area indicates positive FC or co‐activation of these areas, while a negative correlation value indicates negative FC, that is, the time series changes in the opposite direction between these regions. Individual FC maps were converted into *z*‐scored maps with Fisher's *z* transformation to improve normality.

### 2.5 Statistical analyses

Demographic, sleep, mood, cognitive, and physiologic data were examined by Chi‐square (categorical values) and independent samples *t*‐tests (numerical values). We performed one‐sample *t*‐tests on the FC maps to generate positive and negative hippocampus and caudate networks in OSA and control groups (False discovery rate corrections for multiple comparisons [FDR], *p* < .001). We compared the *z*‐scored FC maps between OSA and control subjects using analysis of covariance (ANCOVA; covariates, age and sex; Alphasim corrected, *p* < .05). We examined correlations between regionally‐averaged FC values in brain areas showing abnormal FC in OSA, and AHI, PSQI, ESS, and neuropsychological variables with both Pearson's and partial correlations (covariates: age, gender, and BMI).

## 3 RESULTS

### 3.1 Demographics, sleep, mood, and cognitive variables

No significant differences in age or gender appeared between groups. Both sleep (ESS and PSQI) and mood scores (BDI‐II and BAI) were significantly higher in OSA over control subjects (*p* < .001; Table 1). No significant differences in global MoCA scores emerged between OSA and controls; however, the visuospatial/executive function sub‐scores were significantly lower in OSA from controls (*p* = .009; Table 1).

### 3.2 Hippocampal FC networks

**Figure 1:**
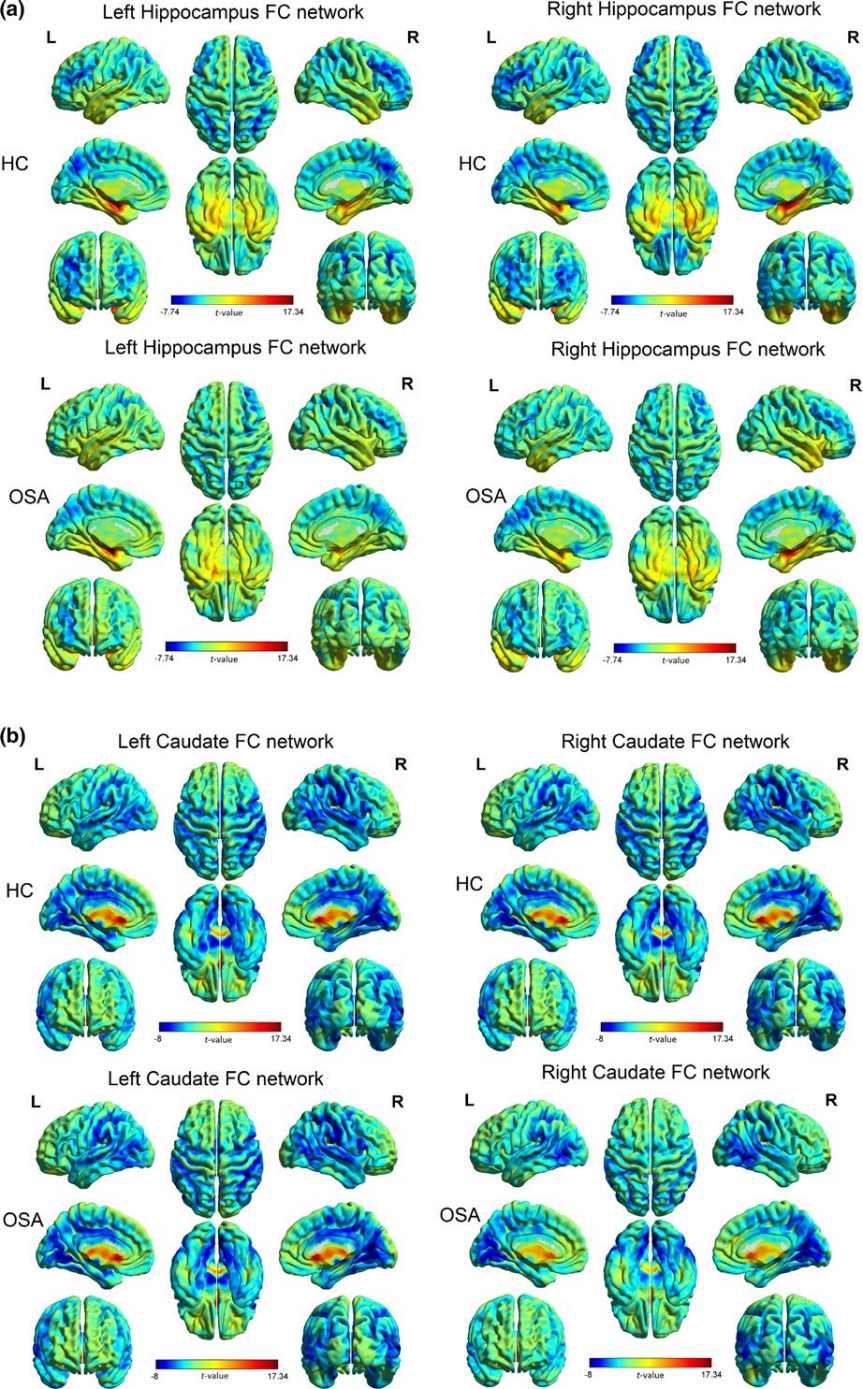
Whole‐brain hippocampal and caudate functional connectivity (FC). (a) Left and right hippocampus showing positive FC (warm color) with the contralateral hippocampal sites, bilateral para‐hippocampal gyrus, insula, thalamus, and superior temporal gyrus in both control (upper row) and obstructive sleep apnea (OSA) (lower row) subjects. Left and right hippocampal regions showing negative FC (cold color) with bilateral middle frontal gyrus, inferior parietal lobule, angular gyrus, and precuneus in both control and OSA. (b) Left and right caudate nuclei showing positive FC (warm color) with the contralateral caudate areas, and with the orbital frontal gyrus in both control (upper row) and OSA (lower row) subjects. Left and right caudate nuclei showing negative FC (cold color) with bilateral mid and posterior cingulate cortices, supplementary motor area, postcentral gyrus, superior temporal gyrus, inferior parietal lobule, angular gyrus, supra marginal gyrus, and ventral tegmentum area in both control and OSA. These maps were generated by performing one‐sample *t*‐tests on the hippocampus and caudate FC maps of healthy controls and OSA patients. Color bar indicates *t* values

In both OSA and control subjects, the left and right hippocampal areas showed positive FC with the contralateral hippocampal sites, bilateral para‐hippocampal gyrus, insula, thalamus, and superior temporal gyrus. The left and right hippocampal regions showed negative FC with bilateral middle frontal gyrus, inferior parietal lobule, angular gyrus, and precuneus. However, compared with the left hippocampus network, the right hippocampus showed stronger negative FC with the rectus and weaker positive FC with left temporal lobe in OSA and control subjects (Figure 1a).

### 3.3 Caudate FC networks

The left and right caudate nuclei in both OSA and control subjects showed positive FC with the contralateral caudate areas, and with the orbital frontal gyrus. The left and right caudate nuclei showed negative FC with bilateral mid and posterior cingulate cortices, supplementary motor area, postcentral gyrus, superior temporal gyrus, inferior parietal lobule, angular gyrus, supra marginal gyrus, and ventral tegmentum area. Compared with the right caudate network, the left caudate showed stronger negative FC with the intraparietal sulcus, middle cingulate cortex, and supplementary motor area in OSA and control subjects (Figure 1b).

### 3.4 Hippocampus FC differences between OSA and controls

**Figure 2:**
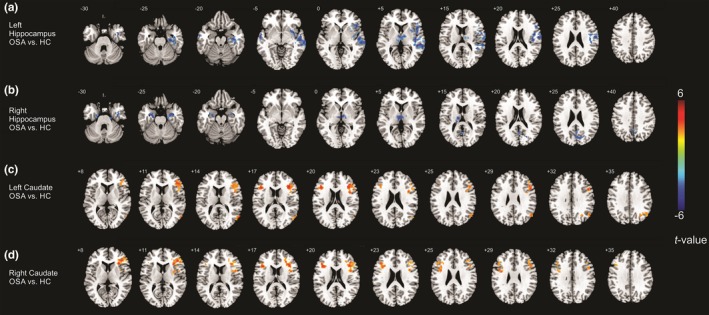
Altered hippocampal and caudate functional connectivity (FC) networks. (a) The left hippocampus showing decreased FC with bilateral medial dorsal thalamus, bilateral medial temporal gyrus, superior temporal gyrus, right anterior and posterior insular, and right para‐hippocampal gyrus in obstructive sleep apnea (OSA) as compared with controls. (b) The right hippocampus showing decreased FC with bilateral medial dorsal thalamus, bilateral hippocampus/para‐hippocampal gyrus, and enhanced negative FC with precuneus/posterior cingulate cortex in OSA over controls. (c) The left caudate nuclei showing altered FC with the bilateral inferior frontal gyrus (IFG), right angular gyrus and inferior parietal lobule in OSA as compared with controls. (d) The right caudate demonstrating altered FC with the bilateral IFG in OSA as compared with controls

In OSA subjects, the left hippocampus showed decreased FC with bilateral medial dorsal thalamus, bilateral medial temporal gyrus, superior temporal gyrus, right anterior and posterior insular, and right para‐hippocampal gyrus (Figure 2a) as compared with controls. While the right hippocampus showed decreased FC with bilateral medial dorsal thalamus, bilateral hippocampus/para‐hippocampal gyrus, enhanced negative FC emerged with precuneus/posterior cingulate cortex (PCC, Figure 2b) in OSA over controls.

### 3.5 Caudate FC differences between OSA and controls

Both the left and right caudate nuclei showed impaired negative FC with the bilateral inferior frontal gyrus (IFG). In addition, the left caudate showed impaired negative FC with the right angular gyrus and inferior parietal lobule (Figure 2c,d) in OSA as compared with controls.

### 3.6 Correlation with behavioral data

**Figure 3:**
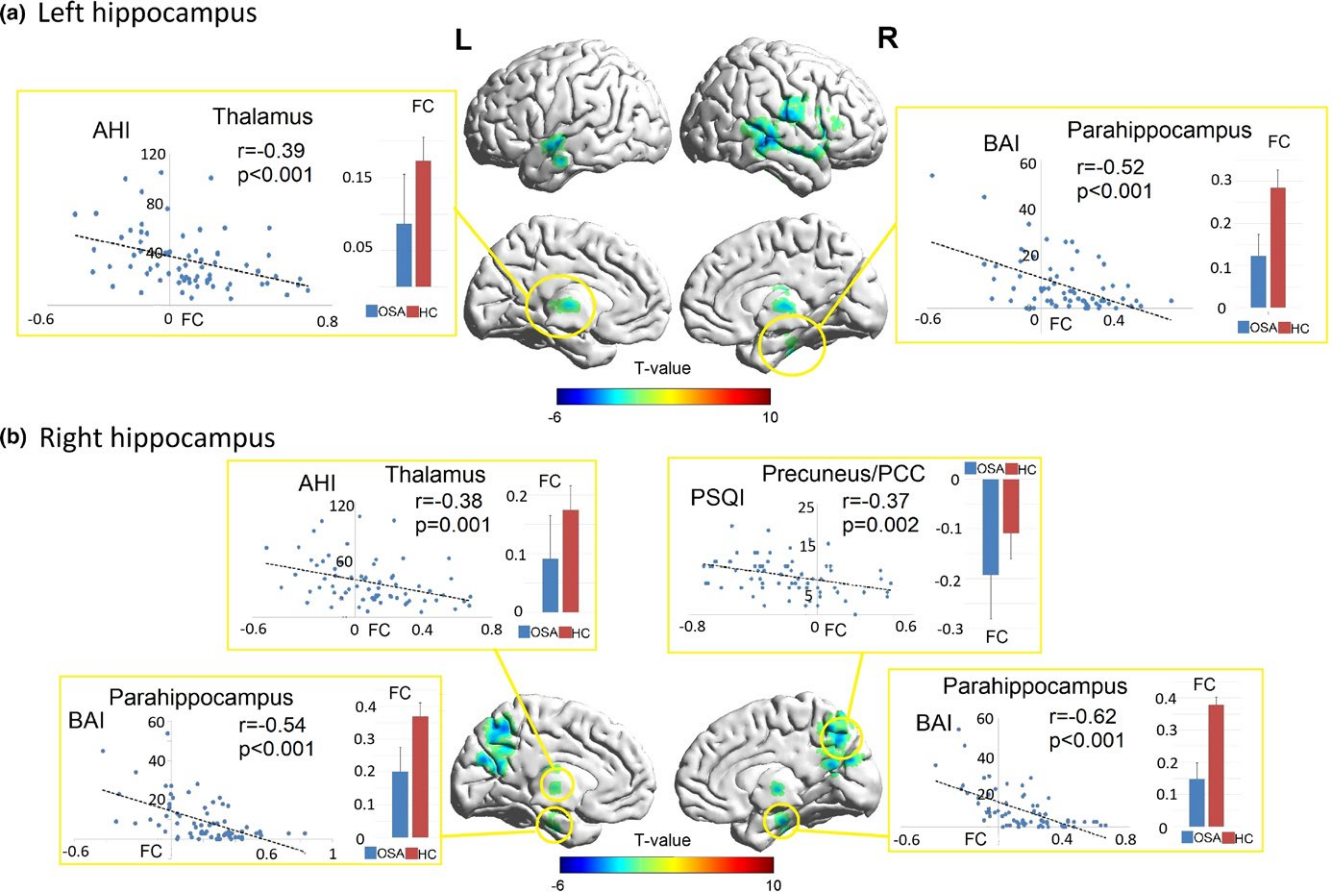
Correlations between altered hippocampus FC with behavioral data. Scatter plots demonstrating relationship between behavioral variables (vertical axis value) and the FC between the seed regions, including left hippocampus (a) and right hippocampus (b), and specific brain areas marked in circle. Blue and red error bar indicate group‐averaged FC values in OSA and control subjects, respectively. AHI, apnea‐hypopnea index; BAI, beck anxiety inventory; FC, functional connectivity; OSA, obstructive sleep apnea; PCC, posterior cingulate cortex; PSQI, Pittsburgh Sleep Quality Index

**Figure 4:**
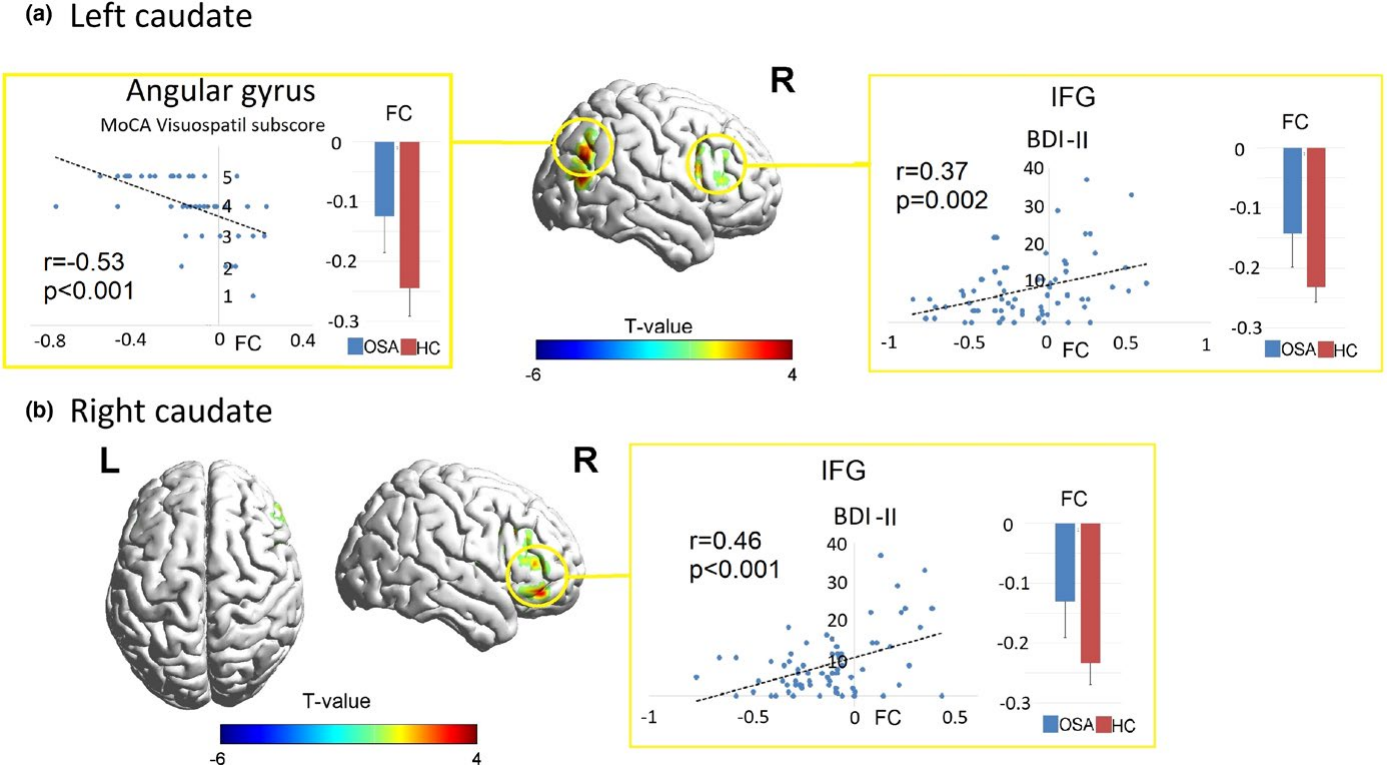
Correlations between altered caudate FC with behavioral data. Scatter plots showing correlation between behavioral variables (vertical axis value) and the FC between the seed regions, including left caudate (a) and right caudate (b), and specific brain areas marked in circle. Blue and red error bar indicate group‐averaged FC value in OSA and control subjects, respectively. BDI‐II, Beck depression inventory II; FC, functional connectivity; MoCA, Montreal cognitive assessment; IFG, inferior frontal gyrus; OSA, obstructive sleep apnea

In OSA subjects, AHI was negatively correlated with decreased FC between the left and right hippocampus and bilateral medial dorsal thalamus. PSQI was negatively correlated with FC between right hippocampus and precuneus. The BAI scores were negatively correlated with decreased FC between left and right hippocampus and bilateral para‐hippocampus gyrus (Figure 3). BDI‐II values were positively correlated with the impaired FC between bilateral caudate nuclei and right IFG. Decreased MoCA visuospatial/executive functional sub‐scores were correlated with impaired FC between left caudate and right angular gyrus (Figure 4). These data are summarized in Table S2. Other brain areas with altered FC did not show significant correlation with any of the behavioral or mood variables.

## 4 DISCUSSION

The objective was to determine how structures essential for mediating principal neuropsychological deficits in OSA interact in resting, baseline states. The data show that both the caudate and bilateral hippocampi demonstrated positive FC to cortical sites that serve mood regulation, awareness, memory, and self‐referential thoughts in both OSA and healthy control subjects. These cortical regions include the medial prefrontal cortex, medial temporal lobe, and angular gyrus. The caudate mainly showed negative FC with cortical areas underlying somatosensory, auditory, visual‐spatial ability, attention, information processing, and executive control. The distinct hippocampal and caudate nuclei functional network patterns suggest that these two subcortical structures are involved in different neurobiological processes, and FC changes from these structures might exert different psychopathological outcomes in OSA. The distribution of positive FC with medial temporal, frontal, and parietal lobes underlie roles in hippocampus‐originated memory and mood regulation, while the widespread negative interactions between caudate nuclei and cortical areas indicate the importance of these structures in sensorimotor gating (Buse, Beste, Herrmann, & Roessner, 2016; Hazlett et al., 2008) and cognitive biasing (Dedovic et al., 2016).

Compared with control subjects, OSA patients consistently demonstrated higher levels of depression and anxiety symptoms and cognitive deficits. We found that these affective symptoms and cognitive issues correlate with impaired hippocampal and caudate FC with multiple brain areas that are involved in mood, executive function, and attention regulation. Intermittent hypoxia, re‐oxygenation, and hyper‐ or hypo‐capnia, along with cerebral blood perfusion changes and sleep fragmentation are common in OSA subjects during sleep. The net effects of these physiological changes on cognition and emotional performance depend on the dynamic stages of both neural adaptive and maladaptive processes, including accumulation of brain injury with time in response to OSA (Rosenzweig et al., 2015; Tahmasian et al., 2016).

We earlier reported brain structural changes in the hippocampus, caudate nuclei, and surrounding white matter in OSA (Kumar et al., 2009, 2012, 2014). The findings here further demonstrate that cognitive and emotional deficits in OSA correlate with abnormal functional interactions between these subcortical structures and cortical areas. Current cognitive theories of mood regulation posit that cognition and emotion are not independent (Mathews & MacLeod, 2005), and our thoughts, inferences, attitudes, and interpretations, as well as the manner in which we attend to and recall information, can predict risk for depression and anxiety (Gotlib & Joormann, 2010; Mathews & MacLeod, 2005). Several mechanisms have been implicated for biased cognitive processing and emotion dysregulation, including inhibitory processes and working memory deficits, ruminative responses to negative mood states and negative life events, and inability to use positive and rewarding stimuli to regulate negative mood. The impaired hippocampal‐cortical FC in OSA may lead to working memory deficits and repetitive rumination of negative mood or events, while the impaired caudate‐cortical FC, as a part of the striatal‐cortical circuits, may indicate reward processing deficits and lack of executive control or inhibition of negative mood (Crane et al., 2016).

### 4.1 Altered hippocampal networks in OSA

Both the left and right hippocampus showed decreased FC with the bilateral medial dorsal thalamus in OSA subjects. The hippocampal role in memory is well described, as is the dorsal thalamus (Squire & Moore, 1979). The thalamus is the object of multiple hippocampal projections, especially through the mammillary bodies, and sometimes described (Stein et al., 2000) as part of the “extended hippocampal system” (Aggleton & Brown, 1999), and the hippocampal‐thalamic FC is crucial for episodic and event memory (Aggleton et al., 2010; Burgess, Maguire, & O'Keefe, 2002). Thalamic connections to the hippocampus and other parts of the mesial‐temporal lobe may differentiate recollective functioning and familiarity memory (Carlesimo, Lombardi, & Caltagirone, 2011). Changes in hippocampal‐thalamic pathways could compromise relay of sensory information to and from the thalamus, and hence impair spatial sensory related episodic memory processing.

The left hippocampus also showed decreased FC with the left insula. Insular cortices are not only involved in somatosensory input integration with interoceptive autonomic action (Oppenheimer, Kedem, & Martin, 1996), but also in reorienting attention and mood regulation. OSA subjects show impaired autonomic regulation and excessive sympathetic tone associated with decreased insular structural integrity (Harper et al., 2003). The insula forms an essential node for processing “relevance” or “salience” in mediating attention and executive action to switch between cognitive, autonomic, pain, and other input in response to relevant stimuli (Kurth, Zilles, Fox, Laird, & Eickhoff, 2010; Menon & Uddin, 2010). The impaired links between the insula and other limbic structures may underlie the switching cognitive resources difficulties from negative rumination to positive stimuli in OSA (Zhang et al., 2015). Aberrant FC between insular cortices and multiple brain regions that play important roles in regulating and/or integrating autonomic, sensorimotor, attention, and affective functions has been described (Park et al., 2016; Zhang et al., 2015). Structural MRI studies also show that damage occurs in the hippocampus and insular cortices in anxious OSA subjects versus non‐anxious OSA, as well as in control subjects, as indicated by prolonged T2‐relaxation values (Kumar et al., 2009). The hippocampus and insular cortices serve affect and autonomic roles, for example, blood pressure changes accompanying emotional dysfunction (Kumar et al., 2009). These sites also show functional deficits to autonomic and respiratory challenges in OSA subjects (Harper et al., 2003; Macey, Kumar, Woo, Yan‐Go, & Harper, 2013). The findings of functional disconnections here between the hippocampus and insula in OSA may affect autonomic regulation, and may further lead to attention, reorientation, and emotional modulation deficits.

We found that both left and right hippocampus showed decreased FC with the contralateral hippocampus/para‐hippocampal gyrus. Decreased interhemispheric coordination between the bilateral hippocampi as well as the mesial temporal lobe, might impair cognitive flexibility in late‐onset subjects with depression (Hou, Sui, Song, & Yuan, 2016). Decreased cognitive flexibility could further affect cognitive‐behavioral regulation of anxiety‐ or depression‐inducing memories (Hou, Sui, et al., 2016; Langenecker et al., 2014). Patients with major depressive disorder showed lower interhemispheric synchrony of the mesial temporal lobe, and demonstrated less efficacy in early therapeutic antidepressants treatment (Hou, Song, et al., 2016). The decreased FC between left and right hippocampus in OSA may lead to less efficacy of cognitive regulation of anxious and depressive thoughts.

The hippocampus also showed decreased FC with the medial temporal lobe in the OSA group. In normal aging individuals, interconnections between the hippocampus and temporal lobe are important for memory formation (Holmes et al., 2014), and interrupted sleep quality heavily influences short‐term memory formation (Chengyang et al., 2016). Reduced hippocampal FC with the temporal regions and frontal lobe occurs following total sleep deprivation; those subjects also demonstrated reduced alertness and decline in short‐term memory performance (Chengyang et al., 2016). The findings here suggest that disrupted hippocampal‐temporal connectivity might be related to emotional memory disturbances in OSA as well (Tahmasian et al., 2016).

The only enhanced FC in OSA over controls appeared between the hippocampus and PCC/precuneus. The PCC is a pivotal node of the default mode network (DMN), which is active when a person is not focused on the outside world and is daydreaming or mind‐wandering (Mason et al., 2007). With multiple structural connections to limbic and para‐limbic cortices, the PCC and precuneus play important roles in combining bottom‐up attention with information from memory and perception; structures are also involved in awareness, arousal, self‐referential thoughts, and remembering the past (Andrews‐Hanna, Smallwood, & Spreng, 2014). The DMN has been reported to be compromised in OSA (Khazaie et al., 2017). The synthesis of findings to date highlights the importance of the posterior DMN, especially the PCC and precuneus, in hyperarousal during sleep and affective symptoms in OSA (Khazaie et al., 2017). The increased negative interaction between hippocampus and PCC and precuneus may indicate more self‐related and retrospective thoughts and less focus on the external world during rest wakefulness in OSA (Khazaie et al., 2017), which in turn, may lead to reduced daytime alertness, decreased vigilance state, and slower reactions to external stimuli (Sforza et al., 2002; Song et al., 2017).

### 4.2 Correlations between altered hippocampal FC and behavioral symptoms in OSA

Decreased FC between the hippocampus and the bilateral medial dorsal thalamus was correlated with higher AHI in OSA. Both the hippocampus and thalamus are sensitive to hypoxia. Chronic intermittent hypoxia decreases NAA/Cr ratios, a reliable measure of neuronal integrity, in both the hippocampus and thalamus of animals (Douglas et al., 2007). Human studies show significant negative correlations between AHI and cerebrovascular reactivity in the thalamus in OSA patients; however, thalamic cerebrovascular reactivity increased with therapeutic positive airway pressure treatment (Prilipko, Huynh, Thomason, Kushida, & Guilleminault, 2014). Diffusion tensor imaging studies also showed significantly decreased white matter fiber integrity in major hippocampal input (cingulum bundle) and output (fornix) pathways (Kumar et al., 2012; Macey et al., 2008). The significant correlations between increased AHI and decreased hippocampal‐thalamic FC found here suggest a close relationship between disease severity and functional deficits in hippocampal and thalamic areas, which may result from loss of structural integrity due to OSA condition (Kumar et al., 2012; Macey et al., 2008).

Furthermore correlation analysis showed that decreased FC between bilateral hippocampus/parahippocampal gyrus was accompanied by increased anxiety symptoms in OSA. The blunted hippocampal activity during positive memory encoding in depression and anxiety disorders may reflect incapacity to disengage from negative emotional or anxious states and engage in more positive ones (van Tol et al., 2012). Hypo‐responses of the hippocampus to positive information may be a generic trait characteristic for both depression and anxiety disorders (Rosenzweig et al., 2015; van Tol et al., 2012). Here, the decreased FC between bilateral hippocampus might indicate higher level of anxiety and memory biases toward negative stimuli in the OSA.

In this study, we found the enhanced negative FC between hippocampus and PCC/precuneus is correlated with higher PSQI scores. This finding suggest that deteriorated sleep quality in OSA may contribute to limbic/para‐limbic circuit dysfunction and lead to drowsiness, excessive mind‐wondering, internally directed or self‐generated thoughts, and less arousal or attention on external stimuli or task during the daytime (Sforza et al., 2002). These data also suggest that comorbid psychological symptoms in OSA might also partially stem from deteriorated sleep quality, daytime sleepiness or drowsiness induced by sleep deprivation accompanying the syndrome (Kahn‐Greene, Killgore, Kamimori, Balkin, & Killgore, 2007; Sforza et al., 2002).

Emerging evidence from human and animal studies indicate that the insular cortex, an important part of the autonomic control and interoceptive system, is a key node of the salience network that generates subjective emotional feelings (Uddin, 2015). Based on functional neuroimaging findings, the insular connectivity with other brain areas are altered in many anxiety disorders (Grupe & Nitschke, 2013), and may contribute to the mediation of intense and persistent fear symptoms as well (Etkin & Wager, 2007). However, such correlations were lacking here. This may be the case that the insula plays multiple roles in autonomic and interceptive information processing, as well as mood regulation. The abnormal FC between the hippocampus and insula in OSA may not be a simple reflection of a specific symptom, but may be a general neural functional deficiency that leads to multiple symptoms, such as autonomic dysregulation, attention reorientation impairments, anxiety, and emotional modulation deficits.

### 4.3 Altered caudate network in OSA

The caudate demonstrated significant negative FC with the IFG and IPL/angular gyrus in the HC; however, these negative FC's were diminished in the OSA group. By interplaying with multiple frontal and parietal cortices, the caudate nuclei play important roles in sensorimotor and visuospatial information gating (Buse et al., 2016; Hazlett et al., 2008) and cognitive attentional biasing (Dedovic et al., 2016). Various studies suggest that regulation of cognitive control is supported by the IFG and inferior parietal regions (Foland‐Ross & Gotlib, 2012). Disruption of this network may contribute to cognitive and mood dysregulation (Langenecker et al., 2014; Silk et al., 2006). Specifically, the IPL and angular gyrus are part of the dorsal visual stream (Rizzolatti & Matelli, 2003), and FC between caudate and the parietal areas of the dorsal visual stream are crucial for space perception, visuospatial function, attention, and action organization (Nagy et al., 2008; Rizzolatti & Matelli, 2003). Impaired functional links between caudate and the parietal areas have been reported to be associated with cognitive and top‐down attentional deficits in several neuropsychological diseases (Diwadkar et al., 2011; Silk et al., 2006). In OSA patients, structural impairments in caudate and posterior parietal cortices have been reported previously (Kumar et al., 2009, 2012, 2014). The abnormal functional connections between bilateral caudate and IPL/angular gyrus may also lead to impaired visuospatial attention and related cognitive functions.

The decreased FC between the caudate nucleus and IFG may underlie the impaired cognitive regulation of emotion, which contributes to comorbid mood disorders in OSA. In the cognitive control model of motivated behavior, the caudate nucleus, as part of the striatum, represents the approach system and underlies motivation, reward processing, and incentive learning (Ernst & Fudge, 2009). Multiple studies reported tight associations between caudate nucleus activation and attentional bias toward positive emotional stimuli, suggesting the capacity of approach‐related cues to engage the caudate and its potential role in reward‐attention association (Lindstrom et al., 2009). The impaired cognitive regulation of emotion in OSA may be reflected by two aspects, both of which are regulated by the striatal‐cortical circuits: decreased sensitivity to reward and attentional bias toward negative information (Ernst & Fudge, 2009). Patients with depression tend to show apathy and anhedonia associated with hypoactivation of fronto‐striatal circuits. The abnormalities in this reward system may lead to a characterization of hypo‐sensitivity to rewards in major depressive disorder, with less interaction between the striatum and frontal areas (Langenecker et al., 2014). Using a decision‐making task, a recent adolescent study with major depressive disorder showed reduced reward‐related activity in the striatal and frontal areas during high‐risk/high‐gain trials (Shad, Bidesi, Chen, Ernst, & Rao, 2011). These studies suggested that the pattern of dysfunction involving decreased striatal‐frontal interaction may be a promising candidate as a depression bio‐marker.

### 4.4 Correlations between altered caudate FC and behavioral issues in OSA

The decreased caudate‐parietal FC was accompanied by decreased visuospatial and executive functions in OSA. Sleep disturbances in OSA subjects can also lead to dysfunction in the attention and cognitive domain (Gupta & Simpson, 2015). Reduced gray matter volume and cortical thickness occur in the IFG and posterior parietal cortex in OSA, which may influence mood and attention processing (Ayalon, Ancoli‐Israel, Aka, McKenna, & Drummond, 2009). Involvement of IPL and angular gyrus participate in voluntary orientating and reorienting of attention, respectively (Corbetta, Kincade, Ollinger, McAvoy, & Shulman, 2000).

The decreased caudate‐frontal FC was correlated with severe depressive symptoms and attention, memory, and attribution biases toward negative information, as well as repetitive negative thoughts, which contribute to depression and anxiety (Mathews & MacLeod, 2005). Specifically, attentional bias toward negative information hinders emotional regulation and employment of positive coping strategies, particularly during periods of challenge or stress induced by decreased sleep quality, and thus, exacerbates the course of depression and anxiety (Gotlib & Joormann, 2010). Other studies examining processing of sad or negative information in depressed samples using several cognitive tasks have also implicated attention control regions as IFG, IPL, and angular gyrus (Foland‐Ross & Gotlib, 2012). The impaired negative interactions between caudate and the bilateral frontal areas may underlie heightened experience of, and an inability to disengage from, negative emotions in OSA.

### 4.5 Limitations

This study has some limitations. The BMI values of controls are not matched with the OSA, since obesity is common characteristic of the condition. Although BMI values were included as a covariate along with age and gender in the additional ANCOVA and partial correlation analyses, no significant relations between BMI and altered brain FC were observed. These findings indicate that functional brain changes found here are most likely the direct effects of OSA condition, instead of obesity. Another limitation is the unavailability of ODI values here. In this study, we have used AHI as an indicator of the overall disease severity level. However AHI is not an accurate index for assessment of hypoxia as ODI. We speculate that the correlation between AHI values and abnormal FC is suggestive of the relationship between disease severity and impaired brain functional integrity. Furthermore relationship studies with ODI are required to confirm that the underlying hypoxia mechanisms are mediating structural and functional brain changes in the condition.

## 5 CONCLUSIONS

In OSA subjects, the hippocampus showed abnormal FC with the thalamus, parahippocampus gyrus, medial and superior temporal gyrus, insular, and precuneus/PCC, and the caudate showed impaired FC with the bilateral IFG and right angular gyrus. These abnormal limbic‐striatal‐cortical FC were accompanied by higher levels of anxiety and depression symptoms, as well as reduced cognitive functioning. The altered hippocampal‐cortical FC may lead to rumination and repetition of negative and anxious mood or events, while the impaired caudate‐cortical FC may indicate deficits in visuospatial functions, reward processing, and absence of executive control or inhibition of negative mood. The altered interactions between brain sites involved in affective and cognitive regulation provide a basis for understanding the comorbidity of cognitive decline and emotional deficits in OSA subjects.

## CONFLICTS OF INTEREST

All authors have no conflicts of interest to declare.
