# Supplementary material for: Altered resting‐state hippocampal and caudate functional networks in patients with obstructive sleep apnea
Source: Brain Behav. 2018 May 10;8(6):e00994. doi: 10.1002/brb3.994 (PMC5991585; doi:10.1002/brb3.994)
Supplement: Supplementary file 1 [file BRB3-8-e00994-s001.docx]

**Supplementary Table 1.** Detailed PSG data and sleep architecture of OSA subjects.

| **PSG Variables** | **Mean** | **Standard deviation** |
| --- | --- | --- |
| SaO_2_ baseline (%) | 95 | 1.9 |
| SaO_2_ nadir (%) | 79 | 8.6 |
| Number of apneas | 23 | 12.5 |
| Number of central apneas | 5 | 7.1 |
| Number of mixed apneas | 4 | 2.7 |
| Number of hypopneas | 56 | 41.2 |
| Sleep efficiency index (%) | 81 | 15.1 |
| Arousal index (number/hour) | 36 | 21.0 |
| Sleep duration (mins) | 358 | 90.8 |
| Wake time (mins) | 98 | 55.8 |
| Stage N1 (mins) | 40 | 25.4 |
| Stage N2 (mins) | 239 | 220.2 |
| Stage N3 (mins) | 19 | 24.5 |
| REM sleep (mins) | 60 | 39.9 |
| Latency of sleep onset (mins) | 13 | 14.1 |
| Wake after sleep onset (mins) | 84 | 50.0 |
| Latency of REM onset (mins) | 155 | 84.1 |
| Number of REM periods | 3 | 1.1 |
| Number of arousals | 180 | 100.5 |

Table legend: SaO_2_, Oxygen saturation; REM, Rapid eye movement;
