# Supplementary material for: Altered resting‐state hippocampal and caudate functional networks in patients with obstructive sleep apnea
Source: Brain Behav. 2018 May 10;8(6):e00994. doi: 10.1002/brb3.994 (PMC5991585; doi:10.1002/brb3.994)
Supplement: Supplementary file 2 [file BRB3-8-e00994-s002.docx]

| ROI | Regions showed altered FC with ROI | Correlated variable | Pearson’s Correlation coefficients | Pearson’s Correlation p-value | Partial correlation coefficients | Partial correlation p-value |
| --- | --- | --- | --- | --- | --- | --- |
| Left hippocampus | Bilateral medial dorsal thalamus | AHI | -0.3883 | <0.001 | -0.3338 | 0.0054 |
|  | right para-hippocampal gyrus | BAI | -0.5215 | <0.001 | -0.5213 | <0.001 |
| Right hippocampus | Bilateral medial dorsal thalamus | AHI | -0.3817 | 0.0011 | -0.3412 | 0.0044 |
|  | Left para-hippocampal gyrus | BAI | -0.5433 | <0.001 | -0.5431 | <0.001 |
|  | Right para-hippocampal gyrus | BAI | -0.6181 | <0.001 | -0.6234 | <0.001 |
|  | Precuneus/PCC | PSQI | -0.3702 | 0.0016 | -0.3568 | 0.0028 |
| Left Caudate | Left Inferior frontal gyrus | BDI-II | 0.3726 | 0.0015 | 0.3864 | 0.0011 |
|  | Left Angular gyrus | MoCA  (Visuospatial) | -0.5288 | <0.001 | -0.5103 | <0.001 |
| Right Caudate | Left inferior frontal gyrus | BDI-II | 0.4571 | <0.001 | 0.4469 | <0.001 |

**Supplementary Table 2.** Correlation between altered brain functional connectivity and behavioral variables. Detailed PSG data and sleep architecture of OSA subjects.

Table legend: SaO_2_, Oxygen saturation; REM, Rapid eye movement; AHI, Apnea-hypopnea index; BAI, Beck anxiety inventory; PSQI, Pittsburgh sleep quality index; BDI-II, Beck depression inventory II; MoCA, Montreal cognitive assessment. Both Pearson’s correlation and partial correlation (adjusted for age, gender, and BMI) coefficients and corresponding p-values are provided.
